# Effects of a probiotic suspension Symprove™ on a rat early-stage Parkinson’s disease model

**DOI:** 10.3389/fnagi.2022.986127

**Published:** 2023-01-18

**Authors:** Marco Sancandi, Carmen De Caro, Neringa Cypaite, Nadia Marascio, Carmen Avagliano, Carmela De Marco, Emilio Russo, Andrew Constanti, Audrey Mercer

**Affiliations:** ^1^Department of Pharmacology, UCL School of Pharmacy, London, United Kingdom; ^2^Department of Science of Health, School of Medicine, University of Catanzaro, Catanzaro, Italy; ^3^Department of Pharmacy, University of Naples Federico II, Napoli, Italy; ^4^Department of Experimental and Clinical Medicine, Magna Græcia University of Catanzaro, Catanzaro, Italy

**Keywords:** Symprove probiotics, gut integrity, short chain fatty acids, Parkinson’s disease, rat model

## Abstract

An increasing number of studies in recent years have focused on the role that the gut may play in Parkinson’s Disease (PD) pathogenesis, suggesting that the maintenance of a healthy gut may lead to potential treatments of the disease. The health of microbiota has been shown to be directly associated with parameters that play a potential role in PD including gut barrier integrity, immunity, function, metabolism and the correct functioning of the gut-brain axis. The gut microbiota (GM) may therefore be employed as valuable indicators for early diagnosis of PD and potential targets for preventing or treating PD symptoms. Preserving the gut homeostasis using probiotics may therefore lead to a promising treatment strategy due to their known benefits in improving constipation, motor impairments, inflammation, and neurodegeneration. However, the mechanisms underlying the effects of probiotics in PD are yet to be clarified. In this project, we have tested the efficacy of an oral probiotic suspension, Symprove™, on an established animal model of PD. Symprove™, unlike many commercially available probiotics, has been shown to be resistant to gastric acidity, improve symptoms in gastrointestinal diseases and improve gut integrity in an *in vitro* PD model. In this study, we used an early-stage PD rat model to determine the effect of Symprove™ on neurodegeneration and neuroinflammation in the brain and on plasma cytokine levels, GM composition and short chain fatty acid (SCFA) release. Symprove™ was shown to significantly influence both the gut and brain of the PD model. It preserved the gut integrity in the PD model, reduced plasma inflammatory markers and changed microbiota composition. The treatment also prevented the reduction in SCFAs and striatal inflammation and prevented tyrosine hydroxylase (TH)-positive cell loss by 17% compared to that observed in animals treated with placebo. We conclude that Symprove™ treatment may have a positive influence on the symptomology of early-stage PD with obvious implications for the improvement of gut integrity and possibly delaying/preventing the onset of neuroinflammation and neurodegeneration in human PD patients.

## 1. Introduction

Parkinson’s disease (PD) is a progressive and debilitating neurological disease with no present cure. For several decades since its discovery, PD was thought to be a brain disorder mainly characterized by both a loss of dopaminergic (DA) neurones in the Substantia Nigra pars compacta (SNpc) and the appearance of aggregates of the protein α-synuclein in the form of Lewy bodies (LBs) leading to a disruption of Ca^2+^ homeostasis, mitochondrial and synaptic dysfunction, oxidative stress, and ultimately cell death (Braak et al., 2003; Poewe et al., 2017; Borghammer and Van Den Berge, 2019). However, Braak and colleagues hypothesized that PD may originate in the gut and spread *via* the vagus nerve to the central nervous system (CNS; Braak et al., 2003) and recent evidence suggests that PD is a multifactorial disease involving a cross talk between the CNS and the peripheral nervous system (PNS; Parashar and Udayabanu, 2017; Tan et al., 2021a). Early signs of gut disruption in PD patients through gastro-intestinal symptoms such as constipation and bloating arising 5–10 years before the onset of motor dysfunction (Yu et al., 2018; Camacho et al., 2021; Metta et al., 2022) and the presence of LBs in the enteric nervous system (ENS) up to 20 years prior to diagnosis (Svensson et al., 2015; Chalazonitis and Rao, 2018) and in human plasma and cerebrospinal fluid (CSF) samples (Concha-Marambio et al., 2019; Magalhaes and Lashuel, 2022) suggest a potential important role of the gut in PD etiology and pathogenesis (Kim et al., 2017; Parashar and Udayabanu, 2017; Dutta et al., 2019; Gazerani, 2019; Castelli et al., 2021).

The health of the gut microbiota (GM) has been directly associated with gut barrier integrity, immunity, function, metabolism and the correct functioning of the gut-brain axis (Rutsch et al., 2020; Morais et al., 2021; Cryan and Mazmanian, 2022; Liu et al., 2022) and their disruption may have a potential role in the etiology of PD (Aho et al., 2021; Romano et al., 2021; Shen et al., 2021; Lubomski et al., 2022). An increased intestinal permeability, presence of gastro-intestinal (GI) inflammation leading to oxidative stress, and changes in microbiota composition in PD patients are thought to correlate with the presence of intestinal α-synuclein and misfolding initiation and neuronal loss (Forsyth et al., 2011; Mulak and Bonaz, 2015; Perez-Pardo et al., 2017a,b; Houser et al., 2018; Lubomski et al., 2020a,b; Aho et al., 2021). Modulation of the microbiota-gut-brain axis (MGBA) may therefore represent a promising therapeutic avenue for the treatment of PD (Dutta et al., 2019; Gazerani, 2019; Castelli et al., 2021; Morais et al., 2021; Liu et al., 2022). Such modulators may include probiotics that are primarily live bacteria naturally occurring in the human gut and delivered in the form of drug, food, or supplements, to alter the composition of the microbiome and exert health benefits to the host (Dutta et al., 2019; Castelli et al., 2021; Tan et al., 2021a). Effects of probiotics have been shown to depend on the strains and the type of disease (McFarland, 2021). However, by colonizing the digestive system, they can boost immune functions and reduce intestinal inflammation (Klaenhammer et al., 2012; Sanders et al., 2019). In recent years, several studies have been conducted to evaluate the effects of probiotics and their potential as a treatment for PD (Dutta et al., 2019; Srivastav et al., 2019; Castelli et al., 2021; Tan et al., 2021a,b; Metta et al., 2022). Probiotic supplementation in PD patients has been shown to improve stool consistency and reduce bloating and abdominal pain (Cassani et al., 2011; Barichella et al., 2016; Knudsen et al., 2017; Dutta et al., 2019; Gazerani, 2019; Srivastav et al., 2019; Castelli et al., 2021; Tan et al., 2021a), improve the MDS-UPDRS (Movement Disorders Society-Unified Parkinson’s Disease Rating Scale) and insulin metabolism (Tamtaji et al., 2019). However, the mechanism of action of probiotics on the gut-brain axis is still not well understood. Symprove™, a probiotic suspension containing four bacterial strains: *Lactobacillus acidophilus* NCIMB 30175, *Lactobacillus plantarum* NCIMB 30173, *Lactobacillus rhamnosus* NCIMB 30174 and *Enterococcus faecium* NCIMB 30176 was shown to be resistant to gastric acidity (Fredua-Agyeman and Gaisford, 2015), to improve symptoms in patients with diverticular disease (Kvasnovsky et al., 2017) and with ulcerative colitis (Bjarnason et al., 2019). As these effects were mainly due to changes in the bacterial composition in the microbiota, production of beneficial short chain fatty acids (SCFAs) and lactate and a beneficial effect on intestinal inflammation (Moens et al., 2019; Ghyselinck et al., 2020), the probiotics suspension was tested in an *in vitro* PD gastrointestinal model resulting in a change in GM composition, improved gut integrity and a decreased gut inflammation (Ghyselinck et al., 2021). In light of these results, the current lack of clinical evidence in PD patients and the limited information available from animal models, we aimed to characterize changes in gut health of an established animal model of early-stage PD in this study and determine the effect of the probiotics on the gut-brain axis.

## 2. Materials and methods

### 2.1. Experimental groups

**Figure 1:**
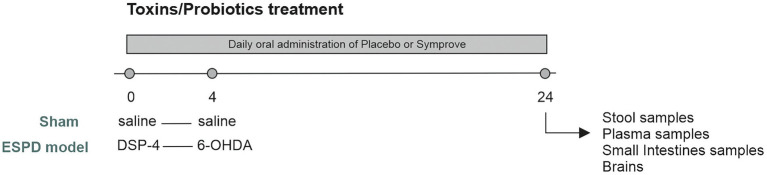
Experimental protocol. Adult male rats (200–250 g) were randomly allocated to four experimental groups. Animals received either saline (Sham) or DSP-4 [Early-stage Parkinson’s Disease (ESPD) model] at Day 0. Three days later, animals underwent stereotaxic surgeries in which they received either saline (sham) or 6-hydroxydopamine (6-OHDA) (ESPD model) and were maintained for 20 days after surgery. 0.5 mL of placebo or Symprove™ were given orally, daily, from day 0 until the end of the experiment. Rats were culled at Day 24 and fecal, small intestine, plasma samples and brains were collected for analysis.

Male albino Wistar rats (P30-40; 200–250 g) were purchased from Charles River Laboratories, United Kingdom. Animals were housed in groups of 4/5 at the UCL School of Pharmacy and had *ad libitum* access to food (Teklad Global 18% Protein Rodent Diet, Envigo) and water. Conditions of humidity (40–60%), temperature (18–22°C) and a 12-h light–dark cycle were kept constant in line with the Home Office regulations. All experiments were approved by the Bloomsbury Animal Welfare and Ethical Review Body (AWERB) and United Kingdom Home Office (PPL PP3144142). Animals were randomly allocated to experimental groups. The early-stage PD rat model was induced as previously described (Sancandi et al., 2018). Briefly, the noradrenergic neurotoxin N-(2-chloroethyl)-N-ethyl-2-bromobenzylamine hydrochloride (DSP-4, 25 mg/kg in saline solution, Sigma Aldrich, Gillingham, United Kingdom) or saline for sham animals were injected intraperitoneally, 4 days prior to stereotaxic striatal bilateral injection of the dopaminergic neurotoxin 6-hydroxydopamine (6-OHDA, 5 mg/mL in saline solution containing 0.9% ascorbic acid, Sigma Aldrich, Gillingham, United Kingdom) or saline for sham animals. The following coordinates were used, from Bregma: AP + 1.0 mm, ML + 3.0 mm, DV-6.5 mm. Each animal received 15 mg of 6-OHDA per striatum (or vehicle) at a flow rate of 1 mL/min^−1^. Both Symprove™ (original flavor) and placebo (matched flavor) were kindly supplied by Symprove Ltd. (Farnham, United Kingdom). 0.5 mL of placebo or Symprove™ (7×10^7^ colony forming units (CFU)/day) was orally delivered daily from Day 1 for 24 days (Figure 1). Placebo (sham and model + placebo) and Symprove™ (sham and Model + Symprove™) (*n* = 4/5 animals per group) experiments were carried out separately to minimize any risk of contamination. The weight of the animals was monitored daily throughout the duration of the experiments. At Day 24, stool samples were collected and frozen. Animals were anaesthetized with inhaled isoflurane (4% v/v in O_2_) followed by an intraperitoneal injection of euthatal (Merial, Harlow, United Kingdom) at a dose of 60 mg/kg. Blood samples were drawn from the heart of anaesthetized animals, and animals were then perfused with ice-cold artificial cerebrospinal fluid (aCSF) containing in mM: 124 NaCl, 25.5 NaHCO_3_, 3.3 KCl, 1.2 KH_2_PO_4_, 1 MgSO_4_, 2.5 CaCl_2_, 15 mM D-Glucose equilibrated with 95% O_2_/5% CO_2_. The ileum (5 cm above the caecum) of each animal from each experimental group was extracted as previously described (Citraro et al., 2021) and frozen for further investigation. Brains were also removed and fixed overnight (4% paraformaldehyde, 0.2% saturated picric acid solution, 0.025% glutaraldehyde solution in 0.1 M phosphate buffer) prior to histological procedures.

### 2.2. Western blot

Small intestine samples were extracted and homogenized in ice-cold lysis buffer [20 mM Tris–HCl (pH 7.5), 10 mM NaF, 150 mM NaCl, 1% Nonidet P40, 1 mM phenylmethylsulfonyl fluoride, 1 mM Na_3_VO_4_, leupeptin and trypsin inhibitor 10 mg/mL; 0.25/50 mg tissue]. After 1 h, tissue lysates were obtained by centrifugation at 20,000 *g* for 15 min at 4°C. Protein concentrations were estimated by the Bio-Rad protein assay (Bio-Rad Laboratories, Milan, Italy) using bovine serum albumin as standard. Small intestine lysate proteins (70 μg) were dissolved in Laemmli sample buffer, boiled for 5 min, and separated by sodium dodecyl sulfate–polyacrylamide gel electrophoresis, and then transferred to a nitrocellulose membrane (240 mA for 40 min at room temperature). The filter was then blocked with 1× phosphate-buffered saline (PBS) and 3% non-fat dried milk for 40 min at room temperature and probed with anti-inducible nitric oxide synthase (iNOS) antibody (dilution 1:1,000; cat. no. 610204, BD Bioscience, Becton Dickinson, Buccinasco, Italy), or anti-cyclooxygenase (COX)-2 (dilution 1:1,000; cat. no. 610431, BD Bioscience,), or anti-NF-κB p65 (dilution 1:500; Santa Cruz Biotechnology, Inc., Santa Cruz, CA, United States), or anti-Iκ-Bα (dilution 1:500; Santa Cruz Biotechnology, Inc.), or anti-occludin (dilution 1:500, cat. no. sc-492, Santa Cruz Biotechnology, at 4°C overnight). The secondary antibody was incubated for 1 h at room temperature. Subsequently, the blot was developed using enhanced chemiluminescence detection reagents (Amersham Pharmacia Biotech, Piscataway, NJ, United States) according to the manufacturer’s instructions. The detection of filter was performed by ChemiDoc Imaging System (Bio-Rad Laboratories). Blots were also incubated in the presence of the antibody against the β actin protein (Sigma-Aldrich, cat.no. G9545, Sigma-Aldrich) to ascertain that they were loaded with equal amounts of protein lysates (De Caro et al., 2020).

### 2.3. ELISA assay

Plasma was obtained from blood collected prior to perfusion (as described above) by centrifugation at 1,000 × *g* for 15 min under 4°C and stored in aliquots at −80°C. The levels of pro-inflammatory cytokines, namely IL-1β, IL-6, TNF-α, and anti-inflammatory cytokine IL-10 as well as bacterial-derived pro-inflammatory lipopolysachharide (LPS), in plasma were measured by ELISA kits (Thermo Fisher Scientific, Monza, Italy) according to the manufacturers’ instructions as previously described (Russo et al., 2014; De Caro et al., 2020; Leo et al., 2020).

### 2.4. Microbial DNA extraction, 16S ribosomal DNA (rDNA) library preparation and sequencing

Freshly evacuated fecal pellets were kept directly in a sterile microtube and stored at −80°C until assayed. Bacterial genomic DNA was extracted from frozen fecal samples using the QIAamp DNA Stool Mini Kit according to manufacturer’s instructions (Qiagen). DNA concentration was measured fluorometrically using a Qubit dsDNA BR assay kit (Invitrogen). Samples were stored at −20°C until processed for amplification. Sequencing samples were prepared according to the protocol 16S Metagenomic Sequencing Library Preparation for Illumina Miseq System with some modifications. The V3–V4 regions of the 16S rDNA gene were amplified and PCR was performed using the following cycle conditions: an initial denaturation step at 95°C for 3 min, followed by 25 cycles of 95°C for 30 s, 55°C for 30 s, 72°C for 30 s and ended with an extension step at 72°C for 5 min. After a purification step with Agencourt AMPure XP (Beckman Coulter Inc), the amplicons were indexed with 10 subsequent cycles of PCR using the Nextera XT Index Kit (Illumina). Each PCR reaction contained 5 μL of amplicons from first PCR, 5 μL index 1 primer (N7xx), 5 μL index 2 primer (S5xx), 25 μL 2× KAPA HiFi HotStart Ready Mix and 10 μL PCR grade water. Library sizes were assessed using an Agilent High Sensitivity 2200 Tape Station System (Agilent Technologies) and quantified with Qubit. Normalized libraries were pooled, denatured with NaOH, then diluted to 10 pM and combined with 25% (v/v) denatured 10 pM PhiX, according to Illumina guidelines. Sequencing run was performed on an Illumina Miseq system using v3 reagents for 2 × 281 cycles (Citraro et al., 2021).

### 2.5. Sequencing data analysis

Paired-end demultiplexed Illumina sequencing reads were imported into the Quantitative Insights Into Microbial Ecology 2 (QIIME 2; 2021.2 distribution)^1^ software suite for downstream analysis (Caporaso et al., 2010). Sequences were then quality filtered, dereplicated, chimeras identified, and paired-end reads merged in QIIME2 using DADA2 (Callahan et al., 2016); quality filtering was performed using default settings, trimming was set at position 60 (forward and reverse), and truncation lengths were set at 240 for forward and reverse, respectively. A phylogenetic tree was generated using the align-to-tree-mafft-fasttree pipeline in the q2-phylogeny plugin. Bray–Curtis dissimilarity between samples was calculated using core-metrics-phylogenetic method from the q2-diversity plugin. Classification of Amplicon Sequence Variants (ASVs) was performed using a naïve Bayes algorithm trained using sequences representing the bacterial V3-V4 rRNA region available from the SILVA database^2^ (Silva_132-99) (Quast et al., 2013), and the corresponding taxonomic classifications were obtained using the q2-feature-classifier plugin in QIIME2. The classifier was then employed to assign taxonomic information to representative sequences of each ASV. Statistical analyses were performed using the Microbiome analyst software.^3^

### 2.6. Fecal SCFA analysis

Fecal samples (*n* = 5 per group) were stored frozen at the end of the experiment. A fixed amount was weighed (0.5 g) and suspended in ultra-pure water (2.5 mL) in a 15 mL tube, vortexed briefly, and then a total of 7.5 mL 1.33% HCl/ethanol solution was added. The samples were homogenized for 2 min and centrifuged at 17,968 *g* for 10 min at room temperature. Supernatant was promptly transferred to a 2 mL sample vial. This step was repeated three times. A quantity of the pooled extract containing acetate, propionate and butyrate were transferred into a 2 mL glass vial and loaded onto an Agilent 7890 gas chromatograph (GC) system with automatic loader/injector (Agilent Technologies, Santa Clara, CA, United States). Pure fatty acid standards (cod. 71251 Acetic Acid, cod. 94425 Propionic Acid, cod. 19215 Butyric Acid Merck Life Science, Milano, Italy) were also prepared to prepare a calibration curve and each sample was analyzed three times on the same day (De Caro et al., 2020).

### 2.7. Immunohistochemistry

Tyrosine hydroxylase (TH) and neuroinflammation immunostainings were carried out on coronal brain sections containing either SNpc or striatum. 50 μm coronal sections were cut using a vibratome (Agar Scientific, United Kingdom). One in every 4 sections containing the SNpc and one in every 12 sections containing the striatum were collected. 4–7 slices were used per animal and per staining. All histological procedures have been previously described (Economides et al., 2018; Sancandi et al., 2018). Sections were incubated in 1% H_2_O_2_ for 30 min and then in 1% sodium borohydride (NaBH_4_) for 30 min to reduce background staining. Sections were then incubated in 10% normal goat serum for another 60 min to prevent non-specific antibody binding. Sections were incubated overnight at 4°C in primary antibodies (Table 1) and 0.1% triton X-100 (Sigma Aldrich). The next day, sections were incubated overnight in secondary antibodies [biotinylated goat anti-mouse or anti-rabbit antibody (1:500 made up in PBS, Vector Laboratories)]. Sections were then incubated in ABC (Vector Laboratories) overnight, washed in TRIS buffer, incubated in 3, 3′ diaminobenzidine (DAB, Sigma Aldrich) for 20 min. Staining was then revealed using H_2_O_2_. All sections were processed using the same immunoreagents and the DAB reaction was stopped by adding TRIS buffer at the same time allowing comparison between experimental groups. Sections were then mounted onto Superfrost slides, dehydrated, cleared with Histoclear and coverslipped using DPX (Sigma Aldrich). Assessors were blinded to the treatment allocation. Specificity of primary antibodies used in this study has been described previously. A SDS-denaturated rat tyrosine hydroxylase purified from pheochromocytoma was used to produce the rabbit polyclonal anti-TH antibody. It reacts with all mammalian species. This antibody specifically recognizes the 60 kDa rat tyrosine hydroxylase (Zhou et al., 1995). Anti-Glial Fibrillary Acidic Protein Antibody, clone GA5 is an antibody against Glial Fibrillary Acidic Protein (GFAP) used to stain astrocytes and Bergman glia cells (Debus et al., 1983). According to the manufacturer’s specifications, in western blots, this antibody labeled a single band at 51 kDa (molecular mass of glial acidic protein). The antibody reacts with GFAP from human, pig, chicken and rat. C-terminus of Iba1 (ionized calcium-binding adapter molecule1) was used for the production of the polyclonal anti-Iba1 antibody that reacts with Iba1 from human, rat and mouse. This antibody stains both resting and activated microglia (Imai et al., 1996; Mori et al., 2000). No staining was observed in sections incubated either with a non-immune serum and secondary antibodies or with primary antibodies only.

**Table 1:** Primary antibodies used for immunoperoxidase staining.

Antibody	Immunogen	Manufacturer/Investigator	Species	Catalogue/Lot number	Dilutions
TH	SDS-denaturated rat tyrosine hydroxylase purified from pheochromocytoma	Sigma	Rabbit	T8700-1VL Lot #SLBL8773V	1:7,500
GFAP	Purified glial filament	Millipore	Mouse	MAB 3402 #2549419	1:7,500
Iba1	C-terminus of Iba1	Wako	Rabbit	019–19741	1:1,000

The optical fractionator probe was used to determine the number of TH-immunopositive neurones in the SNpc (StereoInvestigator, MicroBrightField) using a Nikon microscope coupled to a computer-controlled x-y-z motorized stage and an MBF video camera system. Unbiased stereology was carried out on 5 slices per animal (*n* = 4 per experimental group) with the following parameters: counting frame 70 μm × 50 μm; grid size 175 μm× 175 μm; section thickness 50 μm, dissector height 16 μm. Only neurones with visible nuclei and dendrites were counted.

Levels of GFAP immunohistochemical staining were measured by quantitative thresholding image analysis as previously described (Rahim et al., 2012). Four non-overlapping images of the striatum in each section were captured using a DMR microscope and Leica Application Suite V4 (Leica Microsystems) at X20 magnification with constant light intensity, microscope calibration and video camera settings. Image-Pro Premier (Media Cybernetics, Cambridge, United Kingdom) was used to analyze the images and measure immunoreactivity using a constant threshold that was applied to all images for each respective antigen. Data are presented as the mean percentage area of immunoreactivity ± SEM. Morphological characteristics of Iba1-immunopositive cells (Sholl and branched analyses; *n* = 20 per experimental group) were analyzed using a Neurolucida software (MBF Bioscience). Data are represented as mean ± SEM.

### 2.8. Data analysis

Data were analyzed using GraphPad Prism v.9. The Shapiro–Wilk test and the Kolmogorov–Smirnov test were carried out prior to statistical analysis to determine whether the data followed a normal distribution. The parametric one-way ANOVA test was then used as all data followed a normal distribution.

## 3. Results

### 3.1. Tolerability of Symprove™

**Figure 2:**
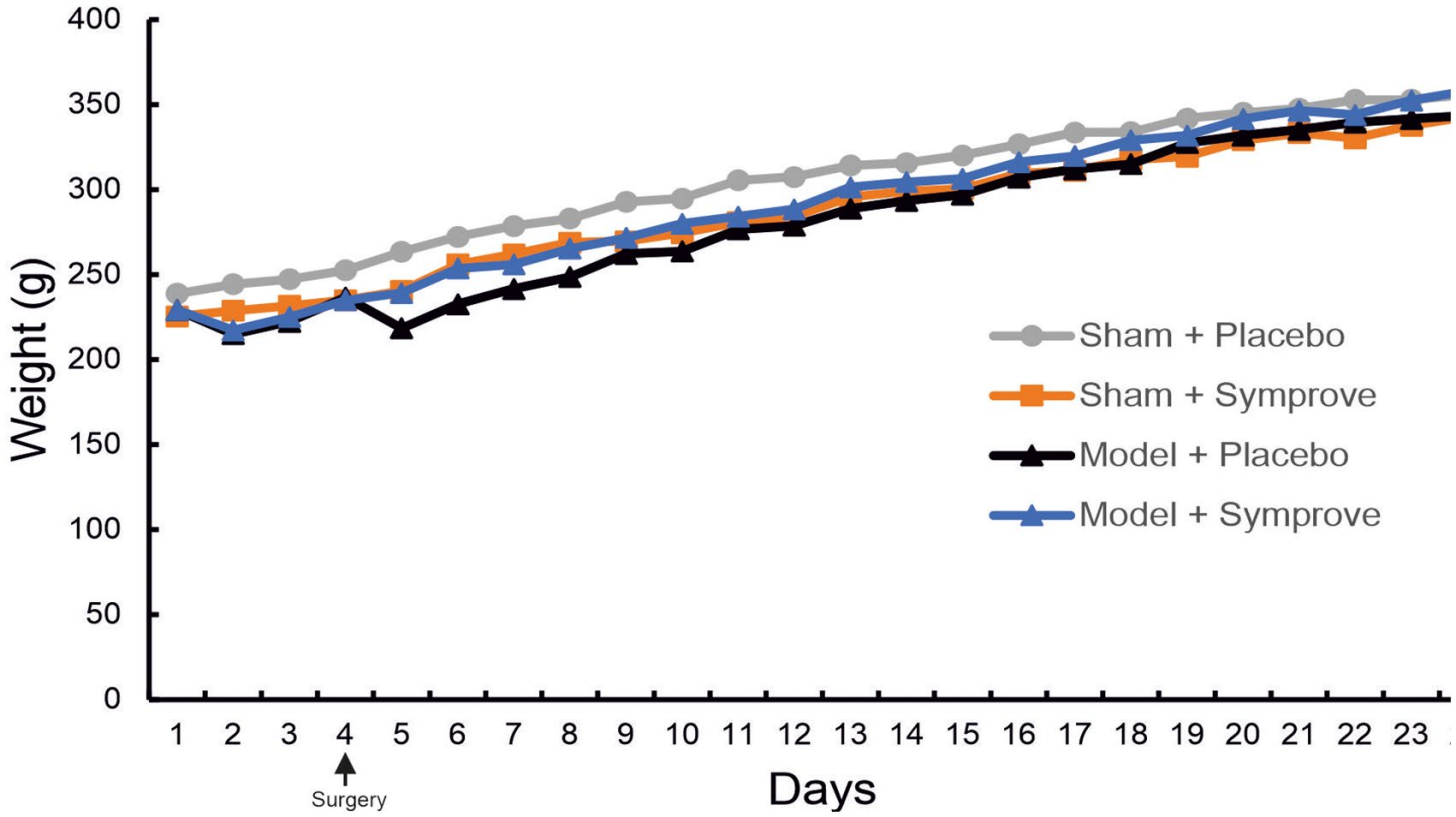
Effect of Symprove™ on animal weight. The weight of animals in the four experimental groups (Sham + placebo, Sham + Symprove™, Model + placebo and Model + Symprove™) was monitored daily from Day 0 until Day 24. Treatment with Symprove™ did not affect the steepness of the growth curve, suggesting a similar weight gain in all experimental groups. Interestingly, PD model animals that received Symprove™ did not display the loss of weight usually observed after the stereotaxic surgery at Day 5.

To assess the tolerability of the probiotics, the weight of the animals was monitored and recorded daily. Animals treated with Symprove™ showed a weight gain comparable to the other groups (Figure 2). Interestingly, rats injected with toxins (PD models) and treated with Symprove™ did not display the characteristic drop in weight following surgery at Day 5.

### 3.2. Symprove™ treatment reduced ileum tissue damage and inflammation observed in the early-stage PD model

**Figure 3:**
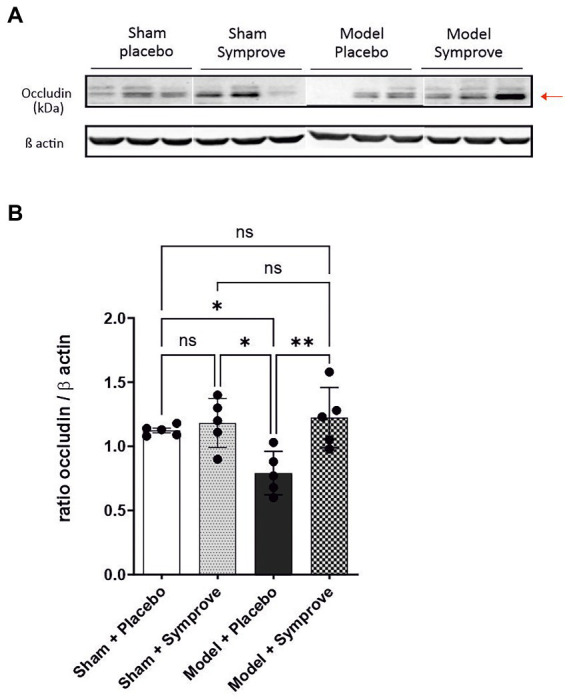
Effect of Symprove™ on gut integrity in the early-stage PD rat model. **(A)** Western blot analysis of occludin levels in the four experimental groups (*n* = 3 per group displayed): Sham + placebo, Sham + Symprove™, model + placebo and model + Symprove™. **(B)** Plots representing occluding/β actin ratio for the four experimental groups (*n* = 5 per experimental group). Data are displayed as mean ± SEM. [ANOVA; ns Not significant, *p < 0.05, **p < 0.01]. The occludin/β actin ratio was decreased in the ESPD model compared with sham animals treated with placebo or Symprove™. This decrease was prevented by treatment with Symprove™.

Expression of occludin in the ileum was measured in the four experimental groups to evaluate intestinal barrier integrity (Figure 3 and Supplementary Figure 1A). Occludin levels measured in the PD model treated with placebo were significantly lower than those in the sham animals treated with placebo and Symprove™ (*p* < 0.05). Symprove™ treatment prevented this decrease (*p* < 0.05).

**Figure 4:**
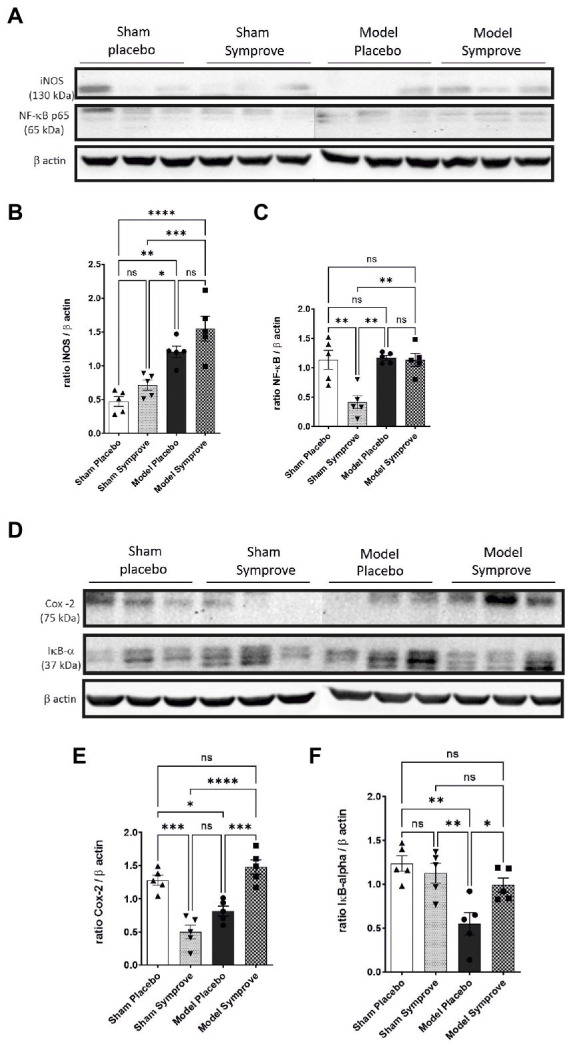
Effect of Symprove™ on inflammatory parameters in the small intestine of the 4 experimental groups (*n* = 3 per group displayed): Sham + placebo, Sham + Symprove™, model + placebo and model + Symprove™. **(A)** Western blot examples of iNOS and NF-κB levels. **(B,C,E,F)** Group comparisons in the expression levels of inflammatory markers denoting an increased inflammatory response in the model group and some prevention by Symprove™ treatment. **(D)** Western blot examples of COX-2 and Iκ-Bα levels. Data are displayed as means ± SEM. *p < 0.05, **p < 0.01, ***p < 0.001, ****p < 0.0001.

The inflammatory state in the ileum of all experimental groups was then assessed by evaluating different pro-inflammatory protein expression, including iNOS, NF-κB, COX-2 and the anti-inflammatory protein Iκ-Bα (Figure 4 and Supplementary Figures 1B,C). iNOS expression was increased in the early-stage PD model compared with that in the sham animals treated with placebo and Symprove^™^ (Figures 4A,B, *p* < 0.05). Expression of iNOS in the PD model animals treated with Symprove^™^ was similar to that in the model animals treated with placebo (*p* > 0.05). Expression of NF-κB in the PD model animals treated with placebo and Symprove™ was similar to that in the sham animals treated with placebo (Figures 4A,C). Surprisingly, sham animals treated with Symprove™ displayed a reduced NF-κB expression compared with the other three experimental groups (*p* < 0.01).

COX-2 expression was significantly decreased in the ileum of PD model animals treated with placebo compared with that in the Sham animals treated with placebo and models treated with Symprove™ (Figures 4D,E, *p*_sham + placebo_ < 0.05; *p*_model + symprove_ < 0.001). Notably, low levels of COX-2 were found after Symprove™ treatment in the sham animals compared with those in the sham animals treated with placebo (*p* < 0.001). IκBα expression was reduced in the model animals treated with placebo compared with that in the sham group treated with placebo and Symprove™ (Figures 4D,F, *p* < 0.01). Symprove™ in the model group prevented this decrease (*p* < 0.05).

### 3.3. Effects of Symprove™ on inflammatory markers in plasma

**Figure 5:**
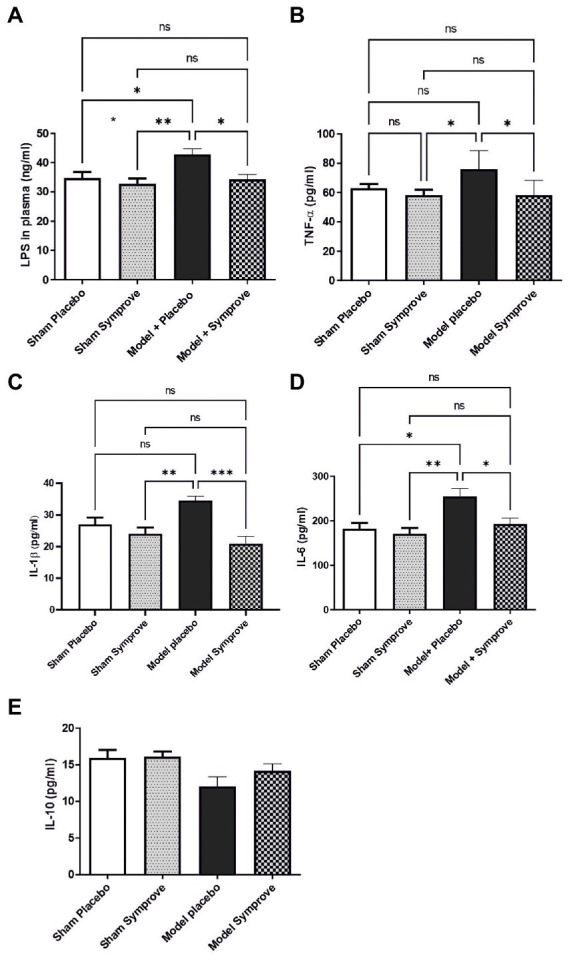
Effect of SymproveTM on inflammatory markers in plasma samples collected from the 4 experimental groups: Sham + placebo, Sham + SymproveTM, model + placebo and model + SymproveTM. Systemic parameters: LPS **(A)**, pro-inflammatory TNF-α **(B)**, IL1β **(C)**, IL-6 **(D)** and anti-inflammatory IL-10 **(E)** cytokine concentrations in plasma analysed by ELISA. Overall, all inflammatory markers were increased by the model and SymproveTM treatment prevented this increase. IL-10, an anti-inflammatory cytokine, was non significantly modified in any group. All data are shown as mean ± SEM. ns, Not significant; *p < 0.05, **p < 0.01, ***p < 0.001.

To investigate the effects of Symprove™ supplementation on inflammatory markers in plasma, the level of lipopolysaccharide (LPS), the pro-inflammatory cytokines IL-6, TNF-α, IL-1β and anti-inflammatory cytokine IL-10 were measured in all experimental groups (Figure 5). An increased level of LPS was observed in the PD model treated with placebo compared with the sham animals (*p*_sham + placebo_ < 0.05; *p*_sham + symprove_ < 0.01) and Symprove™ supplementation significantly decreased its circulating levels (Figure 5A, *p* < 0.05). Plasma levels of IL-6, TNF-α and IL-1β in the PD models treated with placebo were increased compared with those in the Sham animals treated with either placebo and Symprove™ (Figures 5B–D, *p* < 0.05). Treatment with Symprove™ in the PD models prevented this increase (*p*_TNF, IL6_ < 0.05; *p*_IL1_ < 0.001). In addition, animals in the model group treated with placebo exhibited a 25% decrease of IL-10 in plasma compared with the sham groups (Figure 5E). Symprove™ treatment in the model prevented this decrease albeit not significantly.

### 3.4. GM alteration following Symprove™ treatment

**Figure 6:**
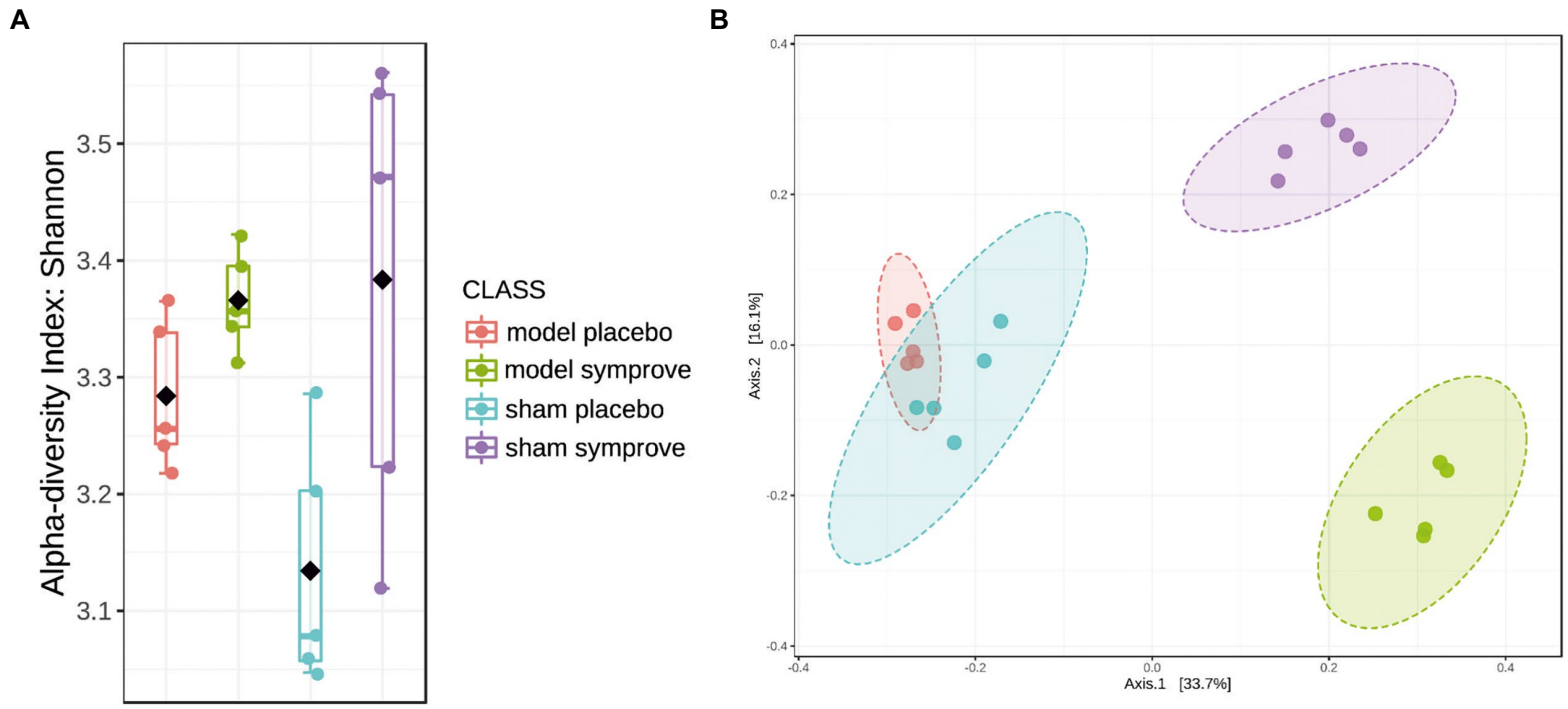
Effect of Symprove™ on gut microbiota structure in all experimental groups (Sham + placebo, Sham + Symprove™, model + placebo and model + Symprove™, *n* = 5 in each group). **(A)** Alpha diversity performed using Shannon Index; Symprove™ treatment was able to increase significantly, alpha diversity both in the sham and model groups. **(B)** Beta diversity was significantly different among all groups.

**Figure 7:**
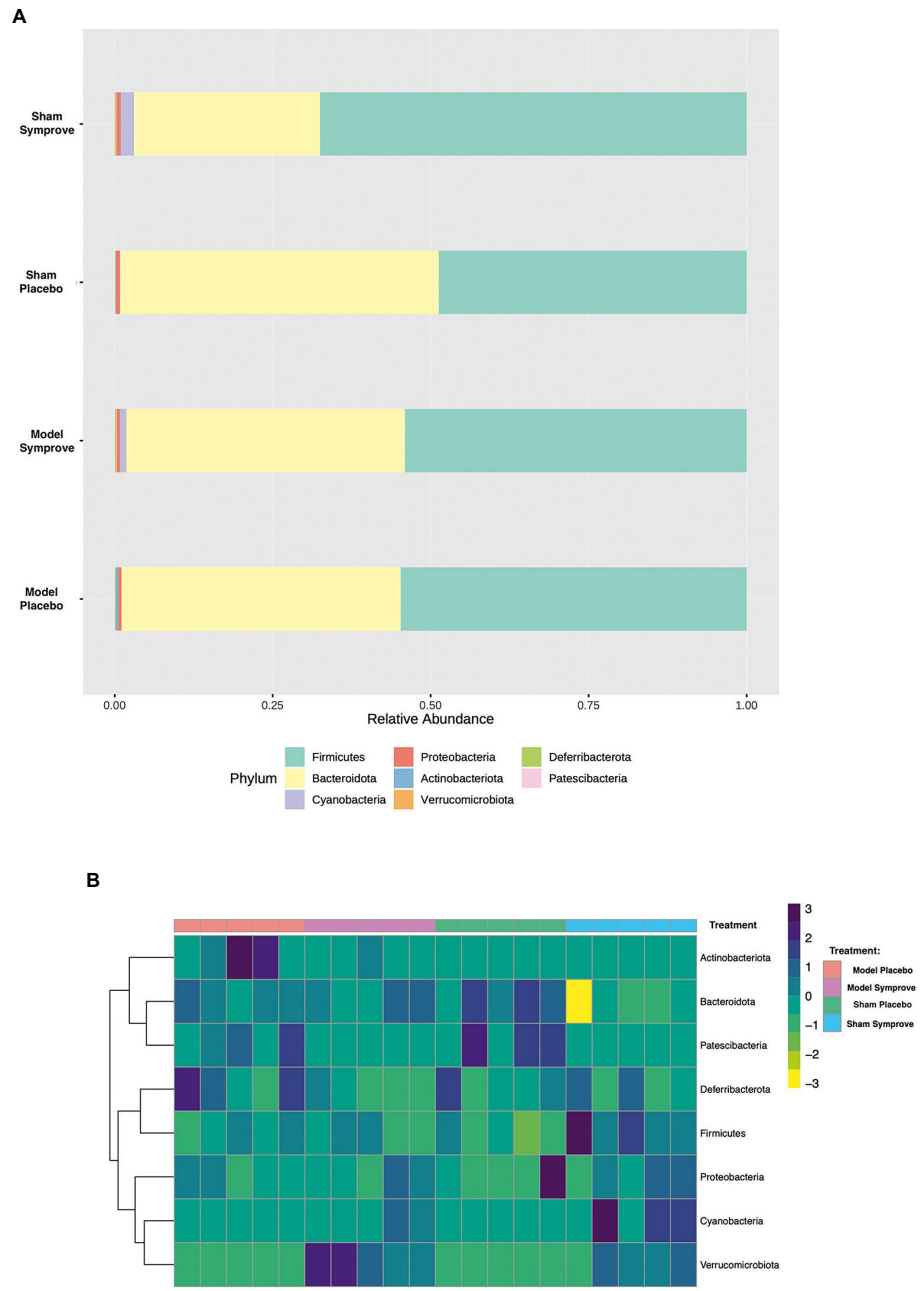
Effect of Symprove™ treatment on bacterial load and the relative abundance at phylum level in all experimental groups. **(A)** Data are shown as relative abundance of the means of a subset of *n* = 5 animals/group; only in the sham group, Symprove™ was able to significantly increase Bacteroidetes. **(B)** Heatmap of most counted operational taxonomic units (OTUs; relative abundance >0.5%) for each sample in the groups.

**Figure 8:**
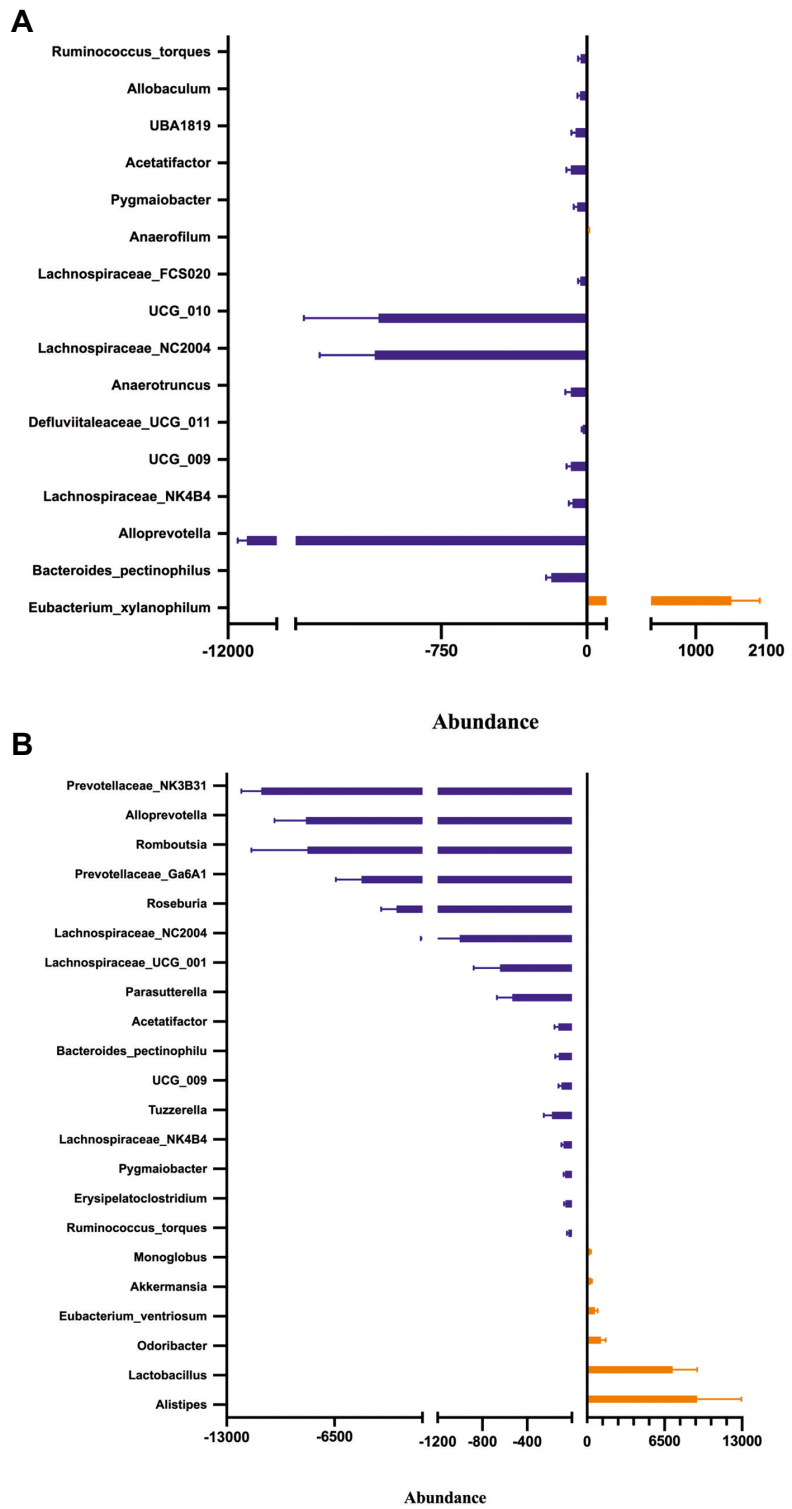
Effect of Symprove™ treatment on bacterial load and relative abundance at genus level. Comparison of relative abundance between Sham + Placebo and Model + Placebo groups **(A)** and between Model + Placebo and Model + Symprove™ groups **(B)**. Only genus differing significantly between the groups were reported, further details are included in Supplementary Tables 1, 2.

Fecal microbiota composition following treatment with either placebo or Symprove™ was analyzed in all experimental groups (Figure 6). The α-diversity results showed that there was a significant difference in the indexes of Shannon among the four groups (Figure 6A). PD model induction was able to significantly increase α-diversity also modifying ß-diversity (Figure 6B); moreover, Symprove™ further increased α-diversity, also significantly modified ß-diversity both in the model and sham control group (Figure 6B). Symprove™ induced a different shift within groups that likely depended on the initial GM composition. GM analysis following treatment with either placebo or Symprove™ reported that, at the phylum level, the predominant bacterial communities were Firmicutes and Bacteroidetes (Figure 7); only in the sham group, Symprove™ was able to significantly increase Bacteroidetes. However, other phylum such as Proteobacteria, Cyanobacteria and Actinobacteria were not significantly modified. On the other hand, several significant differences were evidenced at genus level among groups. PD model induction significantly modified microbiota composition (Figure 8A and Supplementary Table 1); specifically, Eubacterium_xylanophilum were decreased while 15 genera were significantly increased. Notably, among these latter genera, 5 (Acetatifactor, Alloprevotella, Lachnospiraceae_NC2004, Ruminococcus_torques and UCG_009) were reduced by Symprove™ treatment in PD animals (Figure 8B and Supplementary Table 2); furthermore, treatment was overall able to modify 22 genera (Figure 8B and Supplementary Table 2).

### 3.5. Symprove™ supplementation influenced short-chain fatty acid stool expression

**Figure 9:**
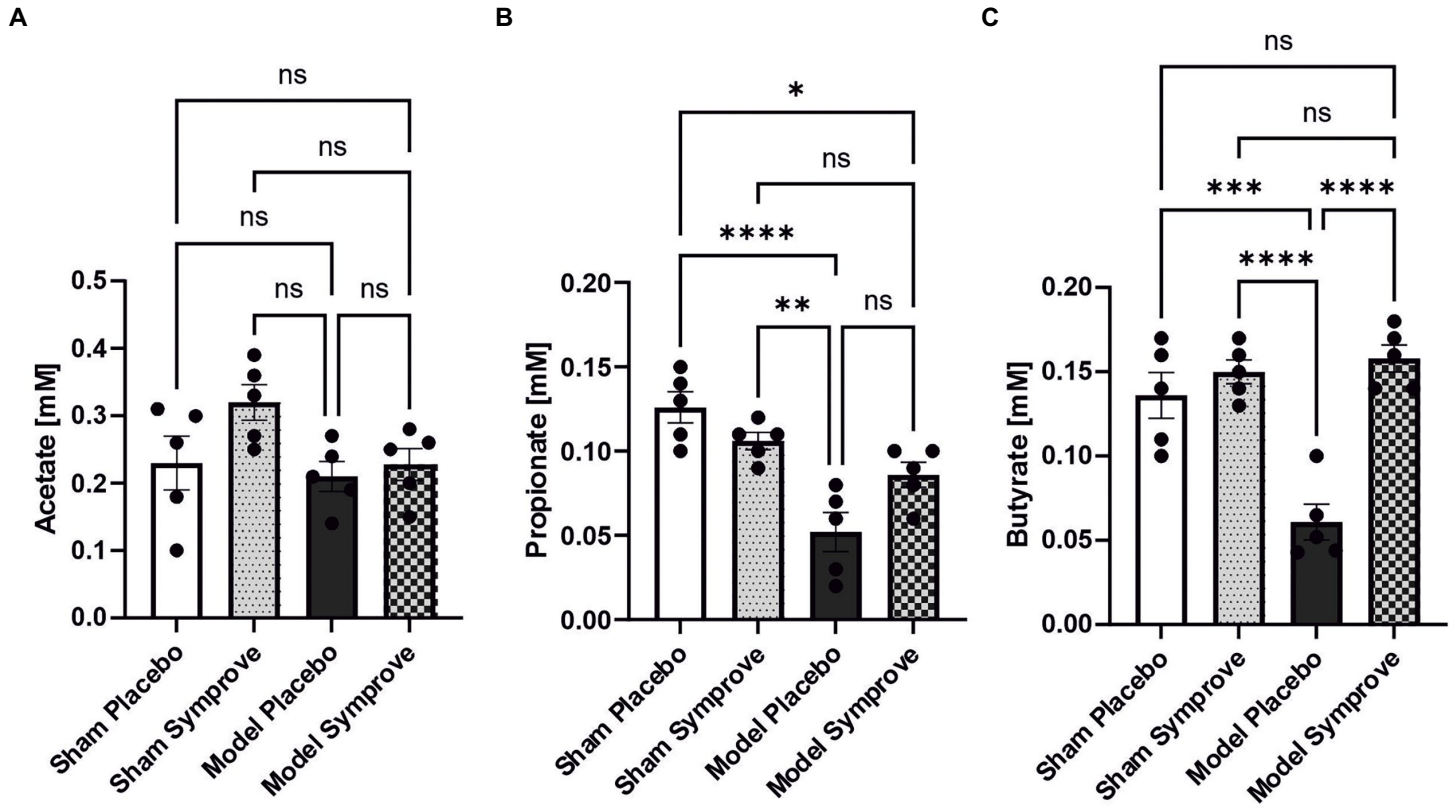
Effect of Symprove™ on short chain fatty acid levels in stool samples collected from all experimental groups: Sham + placebo, Sham + Symprove™, model + placebo and model + symprove™. **(A)** Effect of Symprove™ effects on acetic acid **(A)**, propionic acid **(B)** and butyric acid **(C)** levels in stools. Acetate levels were not significantly modified, while propionate and butyrate levels were reduced in the model and Symprove™ treatment significantly prevented butyrate loss but not completely, the propionate decrease. All data are shown as means ± S.E.M. *p < 0.05, **p < 0.01, ***p < 0.001, and ****p < 0.0001.

Short chain fatty acid concentrations were measured in stool samples from all experimental groups (Figure 9). Acetate levels were similar in all four experimental groups (Figure 9A). Propionate levels were reduced by 60% in the PD model treated with placebo compared with those in the sham animals treated with placebo (Figure 9B – *p* < 0.0001). PD model treated with Symprove™ displayed higher levels than those treated with placebo although this 40% increase was not significant (*p* > 0.05). Levels of butyrate in the PD model treated with placebo were decreased by around 60% compared to those in the sham animals treated with placebo and Symprove™ (Figure 9C – *p*_sham + placebo_ < 0.001; *p*_sham + symprove_ < 0.0001). Treatment with Symprove™ in the model group prevented the decrease in butyrate levels (*p*_model placebo vs. symprove_ < 0.0001).

### 3.6. Treatment with Symprove™ prevented neuroinflammation and decrease in TH-positive cells in the SNpc of the early-stage PD model

**Figure 10:**
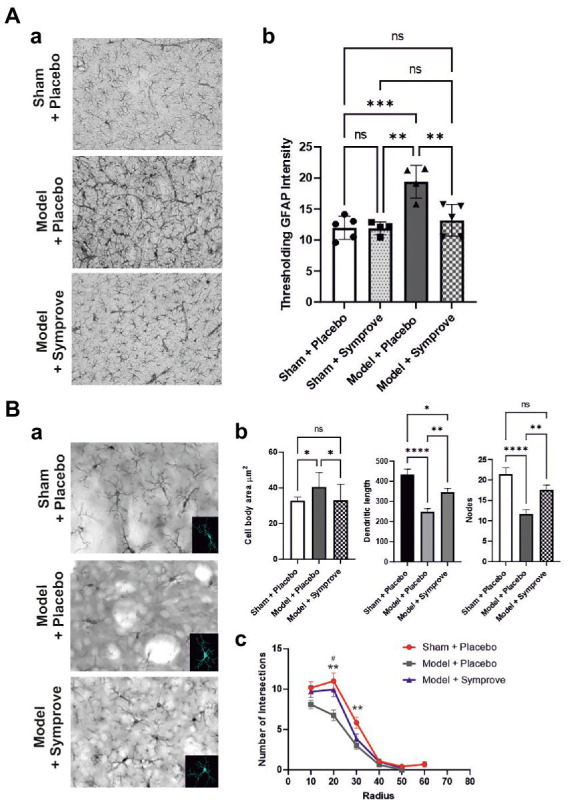
Effect of Symprove™ on striatal neuroinflammation. **(Aa)** Representative immunohistochemical staining of GFAP in the striatum of a sham (top panel) and a PD model animal (middle panel) treated with placebo and a PD model animal treated with Symprove™ (bottom panel). **(Ab)** Thresholding analysis of GFAP staining revealed an increase in the GFAP staining in the PD model treated with placebo compared with that in the sham animals treated with placebo and Symprove™. Symprove™ prevented this increase (ANOVA, *p* < 0.01). Data are presented as mean ± SEM. **(Ba)** Representative immunohistochemical staining of Iba1 in the striatum of a sham animal (top panel) and model (middle panel) treated with placebo and a PD model animal treated with Symprove™ (bottom panel). Insert in each panel represents an example of 3D microglial Neurolucida reconstruction. **(Bb)** Analyses of Neurolucida reconstructions of Iba1-positive cells in the Sham + placebo, Model + placebo and model + Symprove™ groups. Total dendritic length, number of nodes and cell body area of Iba1-positive cells in Sham + placebo, model + placebo and model + Symprove™ groups are represented as mean ± SEM (*n* = 20 per experimental group – ANOVA – *p < 0.05, **p < 0.01, ****p < 0.0001). Treatment with Symprove™ prevented the increase in the size of the microglia cell bodies and partially reduced the decrease in dendritic length observed in the model + placebo (ANOVA, *p* < 0.05). The number of nodes observed in the microglia following Symprove™ treatment was similar to that in the sham + placebo group suggesting more complex dendrites than in the model + placebo (ANOVA, *p*_sham_ > 0.05, *p*_model + placebo_ < 0.01). All data are shown as mean ± S.E.M. **(Bc)** Number of intersections between processes of Iba1-positive cells and concentric sphere at different radius from the soma in the Sham + placebo, model + placebo and model + Symprove™ (Sholl analysis). The number of intersections at 20 μm from the somata of the model + placebo group was significantly smaller than in the Sham + placebo and model + Symprove™ (ANOVA, *p* < 0.01). All data are shown as mean ± S.E.M. ** Sham+placebo vs. Model+placebo *p* < 0.01; # Model+placebo vs. Model+Symprove™, *p* < 0.05, ***p < 0.001.

To determine the effect of Symprove™ on neuroinflammation, levels of GFAP and Iba1 (markers of astrocytic and microglia activation respectively) were assessed in the striatum of the four experimental groups (Figure 10). Astrocytic activation, shown by a stronger GFAP staining, in the PD models treated with placebo was prevented by treatment with Symprove™ (Figures 10Aa,Ab; *p* < 0.01). The change in cell morphology as a result of microglial activation observed in the striatum of the PD model treated with placebo was also prevented by Symprove™ (Figures 10Ba–Bc).

**Figure 11:**
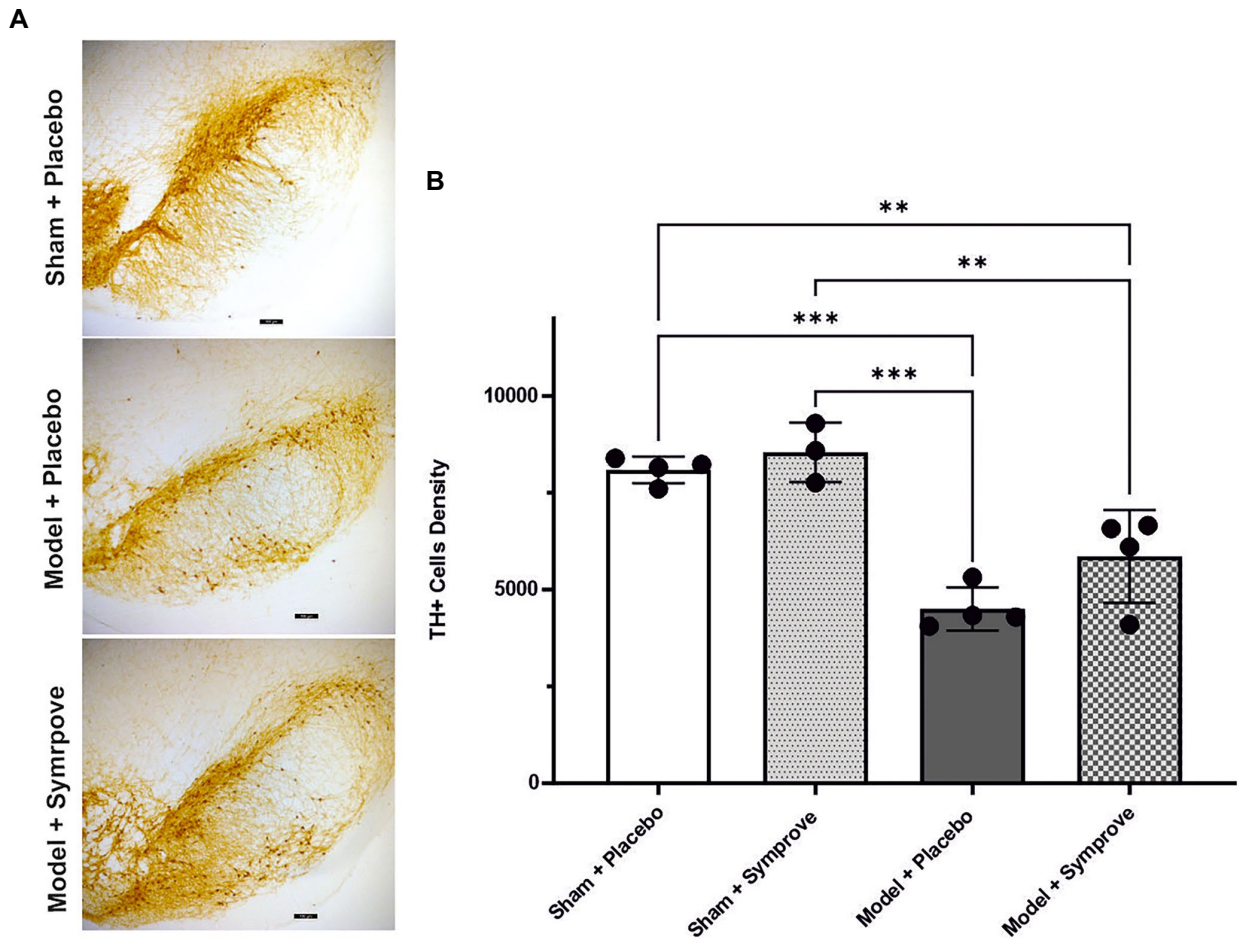
Effect of Symprove™ on neurodegeneration in the SNpc (Substantia Nigra pars compacta) of all experimental groups: Sham + placebo, Sham + Symprove™, model + placebo and model + Symprove™. **(A)** Representative images of TH-staining in the substantia nigra pars compacta (SNpc) in a sham animal and PD model animal treated with placebo and a model treated with Symprove™. Scale bars represent 100 μm. **(B)** Number of TH-positive cells in the SNpc expressed as cell density per mm^3^. A decrease in the number of TH-positive cells was observed in the model treated with placebo compared with the shams treated with placebo and Symprove™ (ANOVA, *p* < 0.001). Symprove™ prevented the loss of TH-positive cells by ~17% compared with models treated with placebo. All data are shown as mean ± S.E.M. **p < 0.01, ***p < 0.001.

Immunohistochemical staining of TH cells in the SNpc (Figure 11A) and unbiased stereology (Figure 11B) revealed that the TH level in the early-stage model treated with placebo was significantly decreased by 45% in the SNpc compared with that observed in sham animals treated with placebo (*p* < 0.001). Treatment with Symprove™ prevented the loss of TH-immunopositive cells in the early-stage model by 17% compared to the model treated with placebo.

## 4. Discussion

A growing body of evidence suggests that the MGBA may play an important role in the etiology of several neurodegenerative diseases including PD (Borghammer and Van Den Berge, 2019; Travagli et al., 2020; Aho et al., 2021; Morais et al., 2021; Wang et al., 2021; Yemula et al., 2021; Tan et al., 2021a; Cryan and Mazmanian, 2022; Dong et al., 2022; Liu et al., 2022). Indeed, disruption of the crosstalk between the GI tract and the CNS linked to gut dysbiosis and inflammation has been shown to influence PD neuropathogenesis in pre-clinical and clinical studies (Houser et al., 2018; Lubomski et al., 2020b; Tan et al., 2021a; Metta et al., 2022). The combination of gut inflammation and deposition of α-synuclein fibrils in the ENS has therefore been suggested to initiate the neuroinflammatory process that leads to a retrograde spread via the vagal nerve to neuronal tissue in the CNS and a potential role in the etiology of the condition (Breid et al., 2016; Ayers et al., 2017; Gazerani, 2019; Lohmann et al., 2019; Van Den Berge et al., 2019; Lubomski et al., 2020b; Castelli et al., 2021; Tan et al., 2021a). In light of these data and the fact that patients diagnosed with irritable bowel syndrome (IBS) have also shown an increased risk of developing PD (Liu et al., 2021), modulators of the gut-brain axis, such as probiotics, may therefore represent a promising therapeutic avenue for PD as they have been shown to improve CNS activity through the modulation of both inflammation and GM (Gazerani, 2019; Castelli et al., 2021; Morais et al., 2021; Liu et al., 2022). However, the mechanism by which probiotics exert their effect remains not well understood. The present study aimed, therefore, at evaluating the effectiveness of supplementation with a probiotic suspension, Symprove™, on both the gut and brain of an early-stage rat model of PD. Although studies have demonstrated host-specific microbiome signatures, most likely due to differences in diet and circadian rhythms, a large proportion of phyla and genera appears to be common in humans and rats (Nagpal et al., 2018) allowing the investigation of the effect of probiotics on the gut-brain axis in a valuable and convenient species. Data shown in this study demonstrated that treatment with the probiotic suspension in the early-stage PD model led to an improved gut integrity and microbiota composition that were correlated with a reduction of gut-derived systemic inflammation and increase in SCFA release. Furthermore, treatment with probiotics prevented striatal neuroinflammation and had a beneficial effect on SNpc dopaminergic cell survival.

### 4.1. Symprove™ treatment improved gut integrity, prevented inflammation, SCFA reduction and LPS elevation by modifying the microbiome in the early-stage PD model

The state of the gut integrity and GM was investigated in the early-stage PD rat model for the first time in this study. As observed in PD patients (Forsyth et al., 2011; Wang et al., 2021), gut permeability was disrupted in the model and a model-dependent modification of the GM composition was observed in terms of several genera although it is important to note that gut dysbiosis in rodent models of 6-OHDA-induced PD remains poorly characterized and often controversial with opposite results being found (e.g., increased/decreased abundance of *Lactobacillus*; Choi et al., 2019; Tsao et al., 2021). As it is often difficult to establish the exact mechanisms underlying the impact of microbiota composition alone on MGBA and the CNS, a recent study assessed common changes in the GM composition observed in studies on PD patients based on the current concept that an alteration of the GM composition and the subsequent imbalance in the MGBA results in increased gut permeability, immune function dysregulation, impaired lipid metabolism and SCFA production contributing to PD pathogenesis (Shen et al., 2021). Although several limitations may be found, this study indicated that PD patients in comparison to healthy controls have a significantly lower abundance levels of Prevotellaceae, Faecalibacterium, and Lachnospiraceae and an increased abundance of the families of Bifidobacteriaceae, Ruminococcaceae, Verrucomicrobiaceae, and Christensenellaceae (Shen et al., 2021). Although comparisons between rats’ and PD patients’ GM compositions cannot be easily translated, our results showed that *Ruminococcaceae* and *Bifidobacteriaceae* were also increased in the model as in PD patients.

Intestinal inflammation, circulating inflammatory markers and SCFA levels were also investigated in the early-stage model in order to determine whether an alteration was present in the model. GM modification was found not to be accompanied, further than by the above reported increased permeability, by a markedly increased inflammatory response in the ileum, at least at this disease stage. In contrast, we found that pro-inflammatory cytokines (i.e., IL-1β, IL-6 and TNF-α) were significantly increased in plasma and the anti-inflammatory cytokine IL-10 was reduced along with an increased concentration of circulating LPS and reduced concentrations of the gut microbial SCFA metabolites butyrate and propionate, considered (at least experimentally) as neuroprotective in other PD models (Ostendorf et al., 2020; Hou Y. et al., 2021; Hou Y. F. et al., 2021). Altogether, these results indicate that gut integrity is damaged in the model and GM is modified to a condition in which SCFA production is disrupted while LPS may either or both be increased in production and/or more easily penetrate into the blood. Similar results have been found in both patients and animal models (Rutsch et al., 2020; Aho et al., 2021; Wang et al., 2021). Association between gut dysbiosis and increased plasma levels of LPS has also been shown in different diseases such as Type 2 diabetes (T2DM), chronic kidney disease (CKD; Salguero et al., 2019), Alzheimer’s Disease (AD; Zhao et al., 2017), inflammatory bowel diseases (IBD; Candelli et al., 2021) and autism (Parracho et al., 2005; Emanuele et al., 2010). However, to our knowledge, elevated blood LPS derived from intestinal dysbiosis in PD subjects has not, so far, been demonstrated. Interestingly, plasma levels of the alternative inflammatory biomarker LPS binding protein (LBP) were found to be consistently lower in PD patients than in healthy patients (Forsyth et al., 2011; Pal et al., 2015; Chen et al., 2021). This inverse correlation between LPS and LBP levels and neuroinflammation in PD patients may have some diagnostic relevance for PD and is worthy of further study (see also Zweigner et al., 2001).

Treatment with the probiotic suspension prevented gut barrier impairment and modified GM composition in the early-stage PD model with 5 genera that were specifically altered by the model being normalized after treatment (e.g., *Acetatifactor, Alloprevotella, Lachnospiraceae_NC2004, Ruminococcus_torques*, and *UCG_009*). Besides this effect, probiotic treatment had a positive impact on circulating cytokines, preventing their alteration by model induction and increasing SCFA levels; this is in agreement with previous observations indicating that probiotic, prebiotic, and synbiotic supplementation may suppress the levels of pro-inflammatory cytokines and decrease the level of NF-ĸB (Romo-Araiza et al., 2018; Tsao et al., 2021). Similarly, SCFA levels were also found to be increased by microbiota manipulation being accompanied by anti-inflammatory and antioxidant effects (Tsao et al., 2021). In line with these effects, the treatment also prevented the LPS increase in plasma. Overall, treatment with the probiotic suspension prevented gut involvement in the model by maintaining a healthier GM, being able to avoid local and systemic inflammation, protecting gut integrity and improving SCFA production.

### 4.2. Symprove™ prevented striatal neuroinflammation and the loss of TH-positive neurones by ~17% in the SNpc

As neuroinflammation is a major player in PD etiology, pathophysiology, and progression of the disease (Cicchetti et al., 2002; Doty, 2012; O'Neill and Harkin, 2018; Arena et al., 2022; Teleanu et al., 2022), the effect of Symprove™ on striatal neuroinflammation in the early-stage PD model was investigated in this study. Supplementation with the probiotics prevented activation of both astrocytes and microglia in the striatum that was observed in the early-stage PD model treated with placebo. This is in agreement with previous studies in which probiotics have been shown to have anti-inflammatory properties (Klaenhammer et al., 2012; Srivastav et al., 2019; Tamtaji et al., 2019; Castelli et al., 2021). Modulation of neuroinflammation was associated with a prevention of the loss of TH-positive neurones in the SNpc of ~17% which was in agreement with recent studies that showed a neuroprotective effect of probiotics in animal models of more advanced stages of PD (Srivastav et al., 2019;Hsieh et al., 2020; Castelli et al., 2021). Castelli and colleagues (2020) demonstrated that treatment with probiotics prevented 6-OHDA-induced neurodegeneration both *in vitro* and *in vivo,* mainly due to anti-inflammatory effects, restoration of pro-survival and neuroprotective pathways and partial prevention of dopaminergic loss. Although the precise mechanisms by which probiotics exert neuroprotective effects are yet to be uncovered, it has been suggested that they may be due to their abilities to both enhance peripheral tyrosine decarboxylase production (van Kessel et al., 2019) and modulate neuroinflammation (Hsieh et al., 2020; Castelli et al., 2021). The early-stage PD model used in this study was shown to display neuroinflammation soon after toxin injection and a reduction in dopaminergic cells in the SNpc (Sancandi et al., 2018) and therefore represents a useful tool to study the etiology of the disease including the cross-talk between the gut and the brain, and to determine new therapeutic targets. Further studies will now be needed to determine the effects of probiotics on PD symptoms (non-motor and motor) and α-synuclein aggregation (in genetic PD models) and to identify their role in modulating the gut-brain axis. Although none of the current animal PD models recapitulates all the symptoms experienced by PD patients exactly, they allow the investigation of the gut-brain axis, how these two organs can influence each other, and which factors are involved in the cascade of events that lead to neurodegeneration.

## 5. Conclusion: Is supplementation with probiotics a new therapeutic avenue for PD treatment?

All current PD therapies aim at mitigating the impact of the symptoms on everyday activities, providing transient relief from the severe deficits. The main PD treatments are focused on both replacing and restoring DA neurotransmission mitigating the motor symptoms, however they are unable to prevent degeneration of the remaining dopaminergic neurones and disease progression. Additionally, current treatments are associated with mild side effects, such as nausea, vomiting, and low blood pressure and more severe side effects, such as behavioral impediments, generation of toxic metabolites and paranoia (Paul and Borah, 2016). Efforts have therefore focussed on using combinations of drugs to reduce the required doses while reducing associated side effects (Baker et al., 2009; Radhakrishnan and Goyal, 2018; Moretti, 2019; Sola et al., 2022). The search for new potential therapies therefore focuses on finding new targets and drugs that will provide a slowing down of disease progression and low side effects, and probiotics are good candidates for effective intervention in the treatment of PD, considering their anti-inflammatory and neuroprotective effects. As the change in the microbiome in the small intestine may reduce drug absorption through gut “leakage” and inflammation and a change in drugs’ pharmacokinetics (van Kessel and El Aidy, 2019), probiotics may potentially act in synergy with standard treatments used in the clinic to slow down the progression of disease and increase the efficacy of current therapies. Data from a double-blind placebo-controlled study of Symprove™ that is currently under way (NCT05146921) will present further insight into the effect of this formulation on gut microbiota and PD symptoms. It would also be of importance to determine whether the probiotics have similar protective effects in patients at different stages of the disease (early-stage vs. more advanced). In addition, further animal studies are needed to determine their benefits in α-synuclein aggregation and their exact mechanism of protective action.

## Data availability statement

The data presented in the study are deposited in the Webin repository (https://www.ebi.ac.uk/ena/browser/home), accession number PRJEB56556.

## Ethics statement

All experiments were approved by the Bloomsbury Animal Welfare and Ethical Review Body (AWERB) and UK Home Office (PPL PP3144142).

## Author contributions

AM, ER, and AC designed the experiments. MS, CDC, NC, NM, CA, CDM, and AM performed the experiments and collected the data. MS, AM, ER, CDC, NC, NM, and CDM performed the analyses. AM and ER drafted the manuscript. All authors read, revised, and approved the manuscript.

## Funding

This study was funded by Symprove Ltd (United Kingdom) and UCL School of Pharmacy internal funds. Symprove Ltd had no role in study design, data collection and analysis, decision to publish or preparation of the manuscript.

## Conflict of interest

The authors declare that the research was conducted in the absence of any commercial or financial relationships that could be construed as a potential conflict of interest.

## Publisher’s note

All claims expressed in this article are solely those of the authors and do not necessarily represent those of their affiliated organizations, or those of the publisher, the editors and the reviewers. Any product that may be evaluated in this article, or claim that may be made by its manufacturer, is not guaranteed or endorsed by the publisher.

## Footnotes

^1^ https://qiime2.org

^2^ https://www.arb-silva.de/download/archive/qiime

^3^ https://www.microbiomeanalyst.ca

## Supplementary material

Supplementary Figure S1 **(A)** Western blot analysis of occludin levels in the four experimental groups: Sham + placebo, Sham + SymproveTM, model + placebo and model + SymproveTM (*n* =2 per experimental group). **(B)** Western blot examples of iNOS and NF-κB levels in the four experimental groups: Sham + placebo, Sham + SymproveTM, model + placebo and model + SymproveTM (*n* =2 per experimental group). **(C)** Western blot examples of COX-2 and Iκ-Bα levels in the four experimental groups: Sham + placebo, Sham + SymproveTM, model + placebo and model + SymproveTM (*n* =2 per experimental group).

## Abbreviations

6-OHDA: 6-Hydoxydopamine
CNS: Central nervous system
COX-2: Cyclooxygenase 2
DSP-4: N-(2-chloroethyl)-N-ethyl-2-bromobenzylamine
ESPD: Early-stage Parkinson’s disease
ENS: Enteric nervous system
GFAP: Glial fibrillary acidic protein
GI: Gastro-intestinal
GM: Gut microbiota
Iba1: Ionized calcium-binding adapter molecule 1
IL: Interleukin
LBs: Lewy bodies
LPS: Lipopolysachharide
LPB: LPS binding protein
MDS-UPDRS: Movement Disorders Society-Unified Parkinson’s Disease Rating Scale
MGBA: Microbiota-gut-brain axis
NF-κB: Nuclear Factor Kappa B
NOS: Nitric oxide synthase
SNpc: Substantia Nigra pars compacta
SCFAs: Short Chain Fatty Acids
TH: Tyrosine hydroxylase
TNF-α: Tumor necrosis factor α
