# Supplementary figures and images for: Effects of a probiotic suspension Symprove™ on a rat early-stage Parkinson’s disease model
Source: Front Aging Neurosci. 2023 Jan 18;14:986127. doi: 10.3389/fnagi.2022.986127 (PMC9890174; doi:10.3389/fnagi.2022.986127)

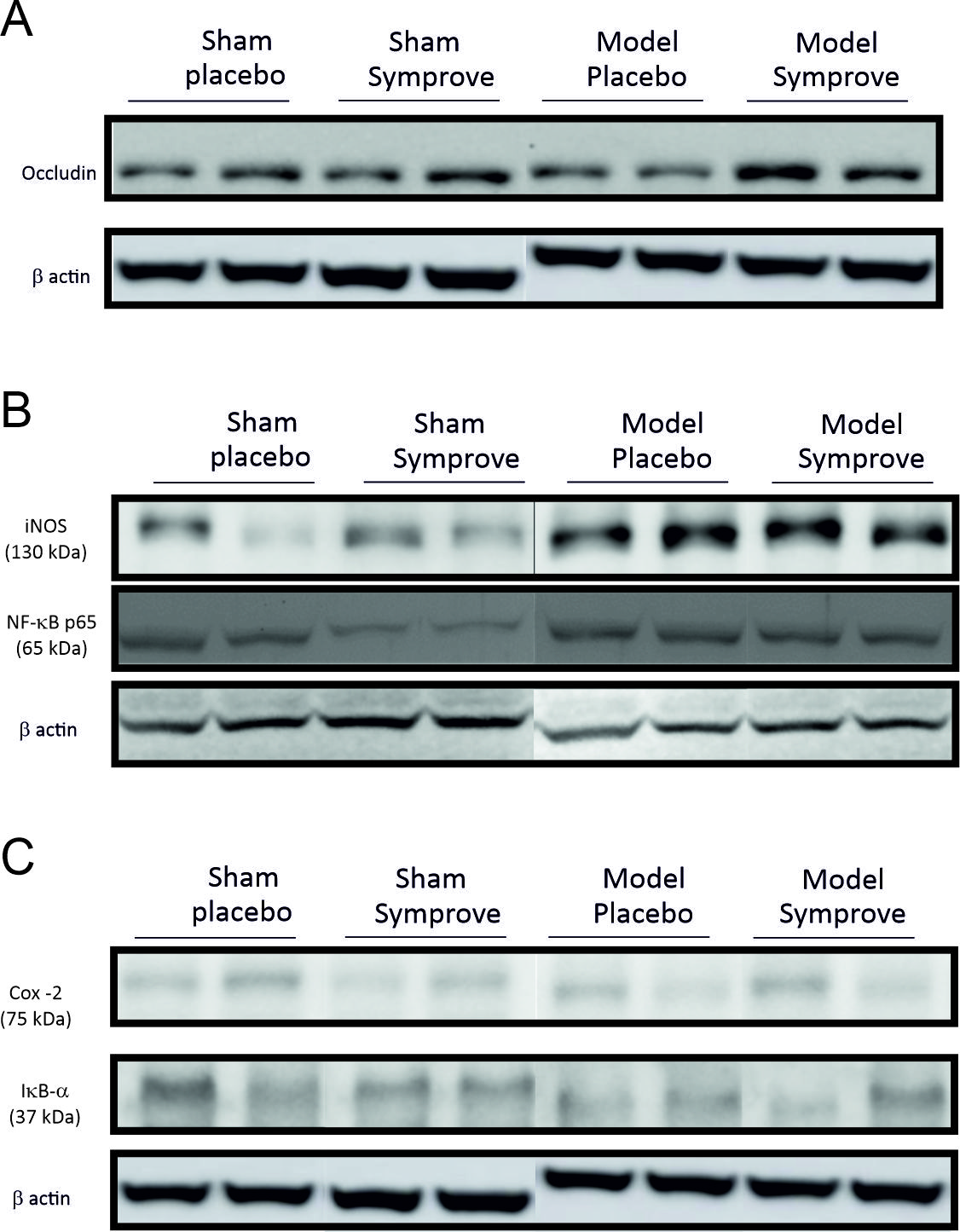

Supplement: Supplementary Figure S1 — (A) Western blot analysis of occludin levels in the four experimental groups: Sham + placebo, Sham + SymproveTM, model + placebo and model + SymproveTM (n =2 per experimental group). (B) Western blot examples of iNOS and NF-κB levels in the four experimental groups: Sham + placebo, Sham + SymproveTM, model + placebo and model + SymproveTM (n =2 per experimental group). (C) Western blot examples of COX-2 and Iκ-Bα levels in the four experimental groups: Sham + placebo, Sham + SymproveTM, model + placebo and model + SymproveTM (n =2 per experimental group). [file Image_1.JPEG]
