# Supplementary material for: Effects of a probiotic suspension Symprove™ on a rat early-stage Parkinson’s disease model
Source: Front Aging Neurosci. 2023 Jan 18;14:986127. doi: 10.3389/fnagi.2022.986127 (PMC9890174; doi:10.3389/fnagi.2022.986127)
Supplement: Supplementary file 2 [file Table_1.DOCX]

**Supplementary Table 1**

**Bacterial genera relative abundance in Sham animals treated with placebo vs ESPD model treated with Placebo.** Significant bacterial genera with relative abundance > 0.5% in at least one of the groups (mean ± SEM) are shown. Variations in genera relative abundances among the groups were assessed by one-way analysis of variance followed by Tukey multiple comparison post hoc test. *p < 0.05, **p < 0.01, ***p < 0.001, ****p<0.0001.

| **Genus** | **Sham + Placebo** | **Model + Placebo** |
| --- | --- | --- |
| Acetatifactor | 70.80 ± 9.140 | 174.4 ± 31.81^**^ |
| Allobaculum | 1600 ±1.600 | 36.80 ± 13.33^*^ |
| **Alloprevotella** | 68.20 ± 32.83 | 8220 ± 1899^**^ |
| Anaerofilum | 9.000 ±2.811 | < LoQ^*^ |
| Anaerotruncus | 15.60 ± 13.92 | 98.40 ± 24.71^*^ |
| Bacteroides_pectinophilus | 1.600 ± 1.600 | 185.2 ± 26.99^***^ |
| Defluviitaleaceae_UCG_011 | 12.80 ± 4.821 | 33.80 ± 2.478^**^ |
| Eubacterium_xylanophilum | 2373 ± 418.2 | 816.4 ± 147.1^**^ |
| Lachnospiraceae_FCS020 | 11.40 ± 7.871 | 45.40 ± 8.778^*^ |
| **Lachnospiraceae_NC2004** | < LoQ | 1092 ± 283.8^**^ |
| Lachnospiraceae_NK4B4 | 2.000 ± 2000 | 75.80 ± 18.89^**^ |
| Pygmaiobacter | 14.80 ± 11.43 | 64.60 ± 13.79^*^ |
| **Ruminococcus_torques** | < LoQ | 32.40 ± 12.97* |
| UBA1819 | 36.40 ± 9.595 | 94.80 ± 18.89^*^ |
| **UCG_009** | 55.80 ± 7.144 | 138 ± 21.47^**^ |
| UCG_010 | 289.2 ± 99.81 | 1361 ± 372.5^*^ |
|  | | |
