# Supplementary material for: Effects of a probiotic suspension Symprove™ on a rat early-stage Parkinson’s disease model
Source: Front Aging Neurosci. 2023 Jan 18;14:986127. doi: 10.3389/fnagi.2022.986127 (PMC9890174; doi:10.3389/fnagi.2022.986127)
Supplement: Supplementary file 3 [file Table_2.DOCX]

**Supplementary Table 2**

**Bacterial genera relative abundance in PD model treated with placebo or Symprove^TM^.** Significant bacterial genera with relative abundance > 0.5% in at least one of the groups (mean ± SEM) are shown. Variations in genera relative abundances among the groups were assessed by one-way analysis of variance followed by Tukey multiple comparison post hoc test. *p < 0.05, **p < 0.01, ***p < 0.001, ****p<0.0001.

| **Genus** | **Model + placebo** | **Model + Symprove** |
| --- | --- | --- |
| Acetatifactor | 174.4 ± 31.81 | 52.60 ± 11.78^**^ |
| Akkermansia | 1.800 ± 1.800 | 330 ± 73.95^**^ |
| Alistipes | 6697 ± 1507 | 15914 ± 3402^*^ |
| Alloprevotella | 8220 ± 1899 | ^**^< LoQ |
| Bacteroides_pectinophilu | 185.2 ± 26.99 | 67.20 ± 16.45^**^ |
| Erysipelatoclostridium | 69.40 ± 11.42 | 11.20 ± 5.361^**^ |
| Eubacterium_ventriosum | 109.2 ± 24.41 | 778.8 ± 214.2^*^ |
| Lachnospiraceae_NC2004 | 1092 ± 283.8 | 89.60 ± 51.88^**^ |
| Lachnospiraceae_NK4B4 | 75.80 ± 18.89 | < LoQ^**^ |
| Lachnospiraceae_UCG_001 | 837.2 ± 213.6 | 194.2 ± 96.86^*^ |
| Lactobacillus | 2965 ± 932.1 | 10151 ± 1822^**^ |
| Monoglobus | 118 ± 35.85 | 372.2 ± 71.18^*^ |
| Odoribacter | 98.60 ± 41.10 | 1253 ± 407.1^*^ |
| Parasutterella | 532.8 ± 138.4 | < LoQ^**^ |
| Prevotellaceae_Ga6A1 | 4874 ± 1541 | < LoQ^*^ |
| Prevotellaceae_NK3B31 | 10916 ± 1207 | < LoQ^****^ |
| Pygmaiobacter | 64.60 ± 13.79 | 3.600 ± 2.205^**^ |
| Romboutsia | 9876 ± 3278 | 1745 ± 849.1^*^ |
| Roseburia | 4591 ± 867.3 | 1835 ± 344.6^*^ |
| Ruminococcus_torques | 32.40 ± 12.97 | < LoQ^*^ |
| Tuzzerella | 233.2 ± 72.78 | 54.80 ± 11.92^*^ |
| UCG_009 | 138 ± 21.47 | 45.60 ± 18.18^*^ |
